# Donor-Derived Cell-Free DNA (dd-cfDNA) in Kidney Transplant Recipients With Indication Biopsy—Results of a Prospective Single-Center Trial

**DOI:** 10.3389/ti.2023.11899

**Published:** 2023-11-03

**Authors:** Louise Benning, Christian Morath, Annette Fink, Markus Rudek, Claudius Speer, Florian Kälble, Christian Nusshag, Jörg Beimler, Constantin Schwab, Rüdiger Waldherr, Martin Zeier, Caner Süsal, Thuong Hien Tran

**Affiliations:** ^1^ Department of Nephrology, Heidelberg University Hospital, Heidelberg, Germany; ^2^ Institute of Immunology, Heidelberg University Hospital, Heidelberg, Germany; ^3^ Institute of Pathology, Heidelberg University Hospital, Heidelberg, Germany; ^4^ Transplant Immunology Research Center of Excellence, Koç University Hospital, Istanbul, Türkiye

**Keywords:** donor-derived cell-free DNA, dd-cfDNA, kidney transplantation, rejection, response to therapy

## Abstract

Donor-derived cell-free DNA (dd-cfDNA) identifies allograft injury and discriminates active rejection from no rejection. In this prospective study, 106 kidney transplant recipients with 108 clinically indicated biopsies were enrolled at Heidelberg University Hospital between November 2020 and December 2022 to validate the clinical value of dd-cfDNA in a cohort of German patients. dd-cfDNA was quantified at biopsy and correlated to histopathology. Additionally, dd-cfDNA was determined on days 7, 30, and 90 post-biopsy and analyzed for potential use to monitor response to anti-rejection treatment. dd-cfDNA levels were with a median (IQR) % of 2.00 (0.48–3.20) highest in patients with ABMR, followed by 0.92 (0.19–11.25) in patients with TCMR, 0.44 (0.20–1.10) in patients with borderline changes and 0.20 (0.11–0.53) in patients with no signs of rejection. The AUC for dd-cfDNA to discriminate any type of rejection including borderline changes from no rejection was at 0.72 (95% CI 0.62–0.83). In patients receiving anti-rejection treatment, dd-cfDNA levels significantly decreased during the 7, 30, and 90 days follow-up compared to levels at the time of biopsy (*p* = 0.006, *p* = 0.002, and *p* < 0.001, respectively). In conclusion, dd-cfDNA significantly discriminates active rejection from no rejection. Decreasing dd-cfDNA following anti-rejection treatment may indicate response to therapy.

**Clinical Trial Registration**: https://drks.de/search/de/trial/DRKS00023604, identifier DRKS00023604.

## Graphical Abstract

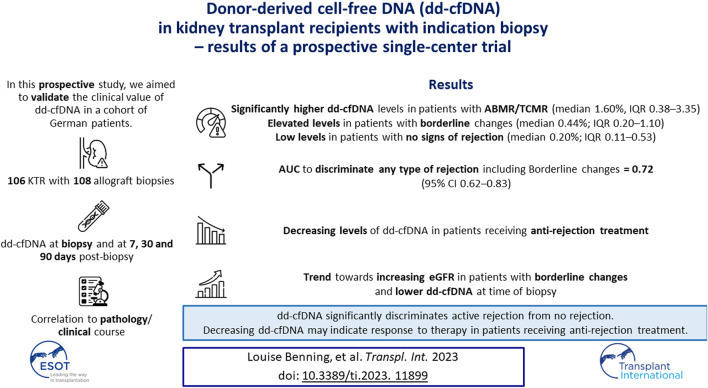

## Introduction

Despite improvements in short-term outcomes after kidney transplantation, mainly driven by improvements in 1 year allograft survival, late allograft failure remains an issue [1, 2]. In a study of 252,910 patients who received kidney transplants in the United States between 1989 and 2009, Lamb et al found that the zero to 1 year rate for graft loss dropped dramatically from 19.8 to 6.7 during this period while rates beyond the first year only showed marginal improvements [3]. Analyzing 108,787 patients from the Collaborative Transplant Study transplanted between 1986 and 2015 and accounting for the evolution of donor and recipient characteristics, Coemans et al found that short-term improvement in more recent years since 2000 was less pronounced, while long-term improvement remained largely unchanged in Europe [4]. Both studies emphasize the pressing need for innovation aimed at improving long-term graft survival.

Meier-Kriesche et al. noted that the limited improvements in long-term allograft survival, despite reduced rejection rates, could be due to acute rejection episodes without complete functional recovery [1], which was supported by results from other clinical trials [5]. Currently, biopsy remains the gold standard for the diagnosis of kidney graft rejection and for the differential diagnosis of kidney graft damage. However, its accessibility is sometimes limited, the right time for biopsy is difficult to determine, and the procedure itself may pose risks, e.g., in obese patients or those requiring anticoagulation. Therefore, there is a need for minimally invasive biomarkers capable of identifying high-risk patients requiring biopsy in the outpatient setting.

In recent years, several advances have been made in follow-up after kidney transplantation, including big data-driven models such as the iBOX to predict allograft survival or new biomarkers such as donor-derived cell-free DNA (dd-cfDNA) to detect early graft damage [6, 7]. Elevated dd-cfDNA levels reflect allograft damage, and studies have shown that dd-cfDNA can effectively distinguish active rejection from no rejection [7–9]. The biomarker was validated in a large US multicenter study of 1,092 kidney transplant recipients over a 3 years period, with an increase in dd-cfDNA to 0.5% or more indicating clinically apparent and subclinical rejection [10]. However, European data on the use of dd-cfDNA is still scarce. In this prospective study, our objective was to analyze dd-cfDNA within a group of German kidney transplant recipients who underwent clinically indicated biopsies, presenting diverse histopathological findings. Our primary aim was to evaluate the sensitivity and specificity of dd-cfDNA in detecting rejection among these patients, and the secondary aim was to explore whether dd-cfDNA levels exhibited changes following anti-rejection therapy, potentially serving as an indicator of treatment response.

## Materials and Methods

### Study Design

From November 2020 to December 2022, we enrolled 106 kidney transplant recipients from the Department of Nephrology at Heidelberg University Hospital with 108 clinically indicated biopsies into this prospective single-center study. The study was approved by the ethics committee of the University of Heidelberg and conducted in accordance with the Declaration of Helsinki. Written informed consent was obtained from all study participants. The study is registered in the German Clinical Trials Register (DRKS00023604).

**FIGURE 1:**
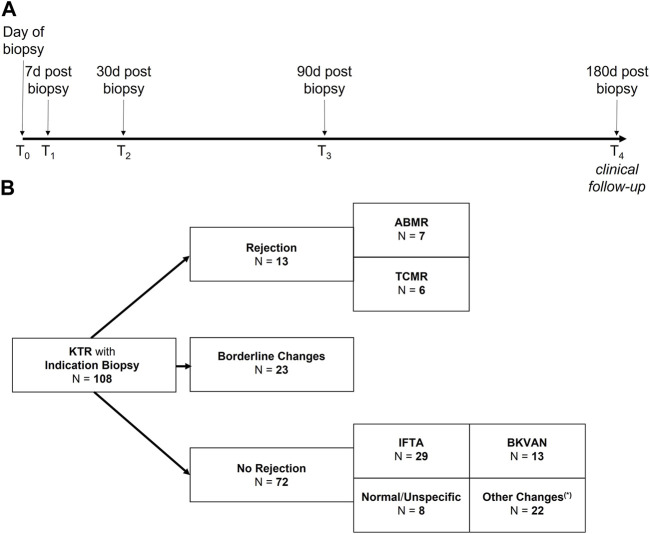
Study design to evaluate the diagnostic potential of donor-derived cell-free DNA (dd-cfDNA) in kidney transplant recipients with indication biopsy. **(A)** Donor-derived cell-free DNA, donor-specific HLA antibodies (DSA) and non-HLA antibodies were determined on the day of biopsy as well as 7, 30 and 90 days post-biopsy. Clinical follow-up was at 180 days post-biopsy. **(B)** Of the 108 allograft biopsies, 36 (33%) were classified as different types of rejection, whereof 7 biopsies were graded as ABMR, 6 as TCMR, and 23 as borderline changes. The 72 biopsies with no signs of rejection were graded either as interstitial fibrosis and tubular atrophy (IFTA, *N* = 29), polyomavirus nephropathy (BKVAN, *N* = 13), normal/unspecific (*N* = 8), or with other changes (*N* = 22). Other changes (*) included acute tubular injury (ATI, *N* = 8), recurrent disease (*N* = 4), infection (*N* = 1), CNI toxicity (*N* = 1), or IFTA with signs of CNI toxicity (*N* = 8). ABMR, antibody-mediated rejection; BKVAN, BK virus-associated nephropathy; CNI, calcineurin-inhibitor; IFTA, interstitial fibrosis and tubular atrophy; KTR, kidney transplant recipients; N, number; T, time point; TCMR, T cell-mediated rejection.

Serum creatinine and the estimated glomerular filtration rate (eGFR), proteinuria, donor-specific HLA antibodies (DSA) and non-HLA antibodies, as well as dd-cfDNA were measured the day of biopsy (before biopsy, T0), as well as 7 (T_1_), 30 (T_2_), and 90 (T_3_) days post-biopsy. Clinical follow-up was until day 180 post-biopsy (T_4_, Figure 1A).

### Indication Biopsy and Clinical Management According to Histopathological Reporting

Indications for biopsy included acute graft dysfunction (*N* = 12), creeping creatinine (*N* = 64) development or worsening of proteinuria (*N* = 16), detection of donor-specific HLA-antibodies (DSA) with concomitant proteinuria or graft dysfunction (*N* = 4), or detection of BK viremia with worsening kidney function (*N* = 12). The biopsy was examined by two board-examined pathologists and reported using the BANFF 2018 reference guide [11]. Following histopathological reporting, clinical management involved corticosteroid pulse therapy in 27/36 (75%) patients with signs of active rejection, including 19/23 (83%) patients with borderline changes and excluding patients with concomitant infection (*N* = 4). In addition, 4/6 (67%) patients with T cell-mediated rejection (TCMR) received anti-thymocyte globulin (ATG) and 3/7 (43%) patients with antibody-mediated rejection (ABMR) immunoadsorption. In all 13 patients with BK virus-associated nephropathy (BKVAN, SV40+), immunosuppression was altered from a calcineurin-inhibitor (CNI)-mycophenolic acid (MPA) to a CNI-mTOR regimen. In 12 patients with suspected CNI-toxicity (ah ≥ 1), CNI medication was adapted to lower trough levels (*N* = 6) or changed to Belatacept (*N* = 6).

### Quantification of Donor-Derived Cell-Free DNA

Venous blood samples were collected into 10 mL Streck cell-free DNA BCT tubes (Streck, Omaha, NE) and examined within 7 days. Plasma was separated by centrifugation at 1,600 × g for 20 min, followed by a second centrifugation at 16,000 × g for 10 min, and either plasma was stored at −80°C or cfDNA was extracted immediately using the Circulating Nucleic Acid kit (Qiagen, Redwood City, CA). cfDNA was then amplified using the AlloSeq cfDNA assay (CareDX, Brisbane, CA), a multiplex PCR including PCR primers for 202 single nucleotide polymorphisms (SNPs). Variations in the SNPs loci are used to determine the proportion of donor-derived (dd)-cfDNA in relation to the total cfDNA present in the plasma sample. The PCR products were subsequently sequenced on a MiniSeq (Illumina, Inc.). Sequence data was analyzed using the CareDx AlloSeq cfDNA software. All steps were performed according to the manufacturers’ instructions and as described previously [12, 13].

### Determination of Donor-Specific HLA Antibodies (DSA) and Non-HLA Antibodies

All patients were screened for DSA and non-HLA antibodies at time of biopsy, as well as 7, 30, and 90 days post-biopsy if serum was available for analysis. Luminex technology was employed to determine HLA antibodies using the LABScreen Single Antigen kit of One Lambda/Thermo Fisher Scientific (West Hills, CA). MFI cutoff of >500 or >1,000 was used to identify DSA against mismatched donor HLA. Testing for non-HLA antibodies included antibodies targeting the major histocompatibility complex class I-related chain A (MICA), angiotensin II type 1 receptor (AT1R) and endothelin receptor subtype A (ETA). MICA antibodies were detected with the LABScreen Mixed kit of One Lambda/Thermo Fisher Scientific (West Hills, CA), whereas AT1R and ETA antibodies were determined with AT1R-IgG-Antibody-ELISA and ETAR-IgG-Antibody-ELISA, respectively (both kits were obtained from CellTrend, Luckenwalde, Germany). Anti-MICA antibodies were found to be associated with ABMR and *de-novo* anti-MICA development was linked to reduced graft survival [14]. AT1R and ETA antibodies were also reported to correlate with a higher prevalence of ABMR and a decline in graft function [15, 16]. Soluble CD30 (sCD30) was assessed using the Human sCD30 Instant ELISA kit of Invitrogen eBioScience/Thermo Fischer Scientific (Bender MedSystems GmbH, Vienna, Austria). Early posttransplant measurements of sCD30 were shown to be predictive of subsequent graft loss, however, the evidence regarding the use of sCD30 as a biomarker in late posttransplant period is limited and its clinical utility remains uncertain [17–19].

### Statistics

Data are presented as number (*N*) and percent (%), median and interquartile range (IQR) or mean and Standard Deviation (SD). Categorical data were compared using the Fisher’s exact test. To compare non-parametric continuous variables between two independent groups, the Mann-Whitney *U* test was used. When dealing with more than two independent groups, the Kruskal-Wallis test was employed, followed by Dunn’s post-test for multiple comparisons. Wilcoxon matched-pairs signed rank test was used when comparing non-parametric paired variables. A multiple linear regression analysis was performed to differentiate possible confounders of elevated dd-cfDNA levels. The area under the ROC curves (AUC) was used to evaluate the performance of dd-cfDNA and eGFR in discriminating acute rejection from no rejection. Rejection status was based on histopathological diagnosis of rejection using the BANFF 2018 reference guide [11]. The Youden index was calculated to give the optimal cut point for dd-cfDNA to discriminate active rejection. In addition, specificity, sensitivity, positive predictive value (PPV) and negative predictive value (NPV) for different dd-cfDNA cutoffs to discriminate acute rejection were calculated using a contingency table. Thresholds of dd-cfDNA levels ≥1%, ≥0.74% and ≥0.5% were applied according to results of the Circulating Donor-Derived Cell-Free DNA in Blood for Diagnosing Active Rejection in Kidney Transplant Recipients (DART) trial [7], early experiences using dd-cfDNA to detect rejection in US American kidney transplant recipients [8], and a recent trial by Stites et al. to identify TCMR1A and borderline patients with elevated risk of graft injury [9], respectively. Spearman’s rho was calculated to assess the correlation between dd-cfDNA levels and histopathological lesion scores or the presence of DSA/non-HLA antibodies. Statistical significance was assumed at a *p*-value < 0.05. The statistical analysis was performed using GraphPad Prism version 9.5.1 (GraphPad Software, San Diego CA, United States). For analysis purposes, serum creatinine and estimated glomerular filtration rate (eGFR) for patients returning to dialysis were arbitrarily set at 10 mg/dL and 5 mL/min, respectively.

## Results

### Baseline Characteristics

From November 2020 to December 2022, 106 kidney transplant recipients with a total of 108 graft biopsies were enrolled. dd-cfDNA was quantified at day of indication biopsy (T_0_), and a median (IQR) of 7 (6–9, T_1_), 38 (28–48, T_2_), and 88 (84–100, T_3_) days post-biopsy. The analytical sample included 370 dd-cfDNA measurements. Clinical follow-up was at a median (IQR) of 185 (172–191) days post-biopsy (T_4_). Patients with a biopsy of <7 days post- transplantation were excluded from analysis.

**FIGURE 2:**
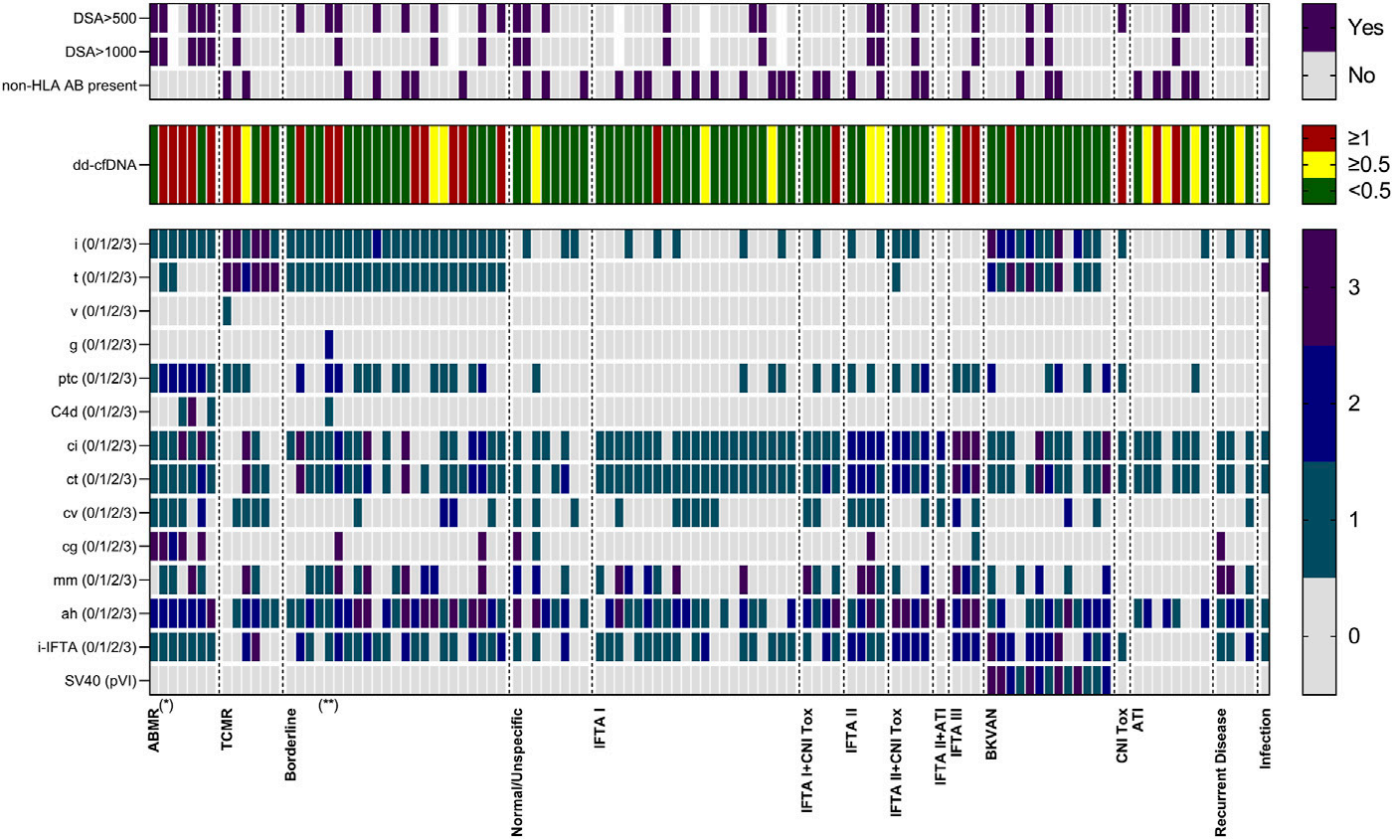
Heat map of histopathological lesion scores according to the BANFF classification and the polyomavirus-associated interstitial nephritis score as well as donor-derived cell-free DNA levels and the presence of donor-specific and non-HLA antibodies for 108 kidney allograft biopsies. The 108 allograft biopsies are grouped according to histopathological diagnosis. Color-coding indicates BANFF lesion scores and dd-cfDNA levels. The presence of donor-specific antibodies with a mean fluorescence intensity of >500 and >1,000, as well as the presence of any non-HLA antibodies is indicated in purple. ABMR, antibody-mediated rejection; ah, hyaline arteriolar thickening; ATI, acute tubular injury; BKVAN, BK virus-associated nephropathy; cg, glomerular basement membrane double contours; ci, interstitial fibrosis; CNI, calcineurin-inhibitor toxicity; ct, tubular atrophy; cv, vascular fibrous intimal thickening; dd-cfDNA, donor-derived cell-free DNA; DSA, donor-specific antibodies; g, glomerulitis; i, interstitial inflammation; IFTA, interstitial fibrosis and tubular atrophy; mm, mesangial matrix thickening; non-HLA AB, non-HLA antibodies (angiotensin II type 1 receptor/endothelin receptor subtype A/major histocompatibility complex class I-related chain A); ptc, peritubular capillaritis; PVI, polyomavirus-associated interstitial nephritis score; t, tubulitis; v, intimal arteritis; TCMR, T cell-mediated rejection; i-IFTA, inflammation in the area of IFTA. (*) Two biopsies showed mixed rejection with concomitant borderline lesions and were categorized as ABMR due to low numbers of mixed rejections. (**) Based on clinical judgement, this biopsy was categorized as borderline changes, despite the presence of glomerulitis, peritubular capillaritis, C4d deposition, and low-level DSA (MFI 505). Of note, the biopsy was conducted 14 days after a living kidney donation, DSA were not detected subsequently and eGFR as well as dd-cfDNA improved upon sole corticosteroid treatment.

Of the 108 allograft biopsies, 36 (33%) were classified as different types of rejection, whereof 7 biopsies were graded as ABMR, 6 as TCMR, and 23 as borderline changes (Figure 1B). Subcategories of ABMR and TCMR with respective dd-cfDNA levels are given in Supplementary Table S1. The 72 biopsies with no signs of rejection were either graded as interstitial fibrosis and tubular atrophy (IFTA, *N* = 29), polyomavirus nephropathy (BKVAN, *N* = 13), normal/unspecific (*N* = 8), or with other changes (*N* = 22, Figure 1B). Figure 2 displays dd-cfDNA levels, the presence of DSA at an MFI cutoff >500 or >1,000, the presence of any non-HLA antibodies determined, and corresponding histopathological lesions for each biopsy.

Patient characteristics stratified for active rejection vs. no rejection are shown in Table 1. Since no protocol but only indication biopsies in the presence of allograft dysfunction had been performed, patients with borderline changes were included into the active rejection group. No statistically significant differences in sex or age were seen between patients with rejection and those without (*p* > 0.99 and *p* = 0.1, respectively). Patients with active rejection had significantly higher levels of proteinuria (*p* = 0.002), and were more likely to be DSA+, albeit without reaching statistical significance (*p* = 0.07 for DSA with MFI >500, *p* = 0.31 for DSA with MFI >1,000, Table 1).

**TABLE 1:** Clinical characteristics in kidney transplant recipients with indication biopsy stratified for status of rejection.

Variable	Active rejection	No active rejection	*p-*value
Number of Samples, *N* (%)	36 (33)	72 (67)	
Female, *N* (%)	12 (33)	23 (32)	>0.99
Age at enrollment, Median (IQR)	43 (34–62)	54 (39–62)	0.11
Donor type			**0.003** (**)
Deceased donor, *N* (%)	16 (44)	54 (75)	
Living donor, *N* (%)	20 (56)	18 (25)	
HLA-A+B mismatches, Median (IQR)	2 (1–3)	2 (1–2)	0.28
HLA-DR mismatches, Median (IQR)	1 (0–1)	1 (0–1)	0.16
Months post-transplant at time of biopsy, Median (IQR)	36 (3–135)	28 (3–72)	0.67
DSA MFI > 500, *N* (%)	14 (41)^a^	16 (23)^b^	0.07
DSA MFI > 1,000, *N* (%)	9 (26)^a^	12 (17)^b^	0.31
Presence of non-HLA AB, *N* (%)	7 (19)	28 (39)	0.05
sCD30 > 40, *N* (%)	11 (31)	12 (17)	0.13
S-Creatinine [mg/dL], Median (IQR)	2.5 (1.7–3.2)	2.2 (1.8–3.3)	0.84
eGFR [mL/min/1.73 m^2^], Median (IQR)	26.8 (20.6–43.0)	28.3 (17.4–38.8)	>0.99
Proteinuria [g/molCr], Median (IQR)^c^	100.4 (46.4–223.3)	35.6 (17.5–113.4)	**0.002** (**)
dd-cfDNA [%], Median (IQR)	0.6 (0.2–1.7)	0.2 (0.1–0.5)	**<0.001** (***)

The data includes two patients with re-biopsies after completed follow-up. dd-cfDNA, donor-derived cell-free DNA; DSA, donor-specific anti-HLA antibodies; non-HLA AB, non-HLA antibodies including antibodies targeting the major histocompatibility complex class I-related chain A (MICA), angiotensin II type 1 receptor (AT1R) and endothelin receptor subtype A (ETA); ***p < 0.001; **p < 0.01.

^a^ Not possible to determine DSA in two patients due to missing data.

^b^ Not possible to determine DSA in three patients due to missing data.

^c^ Data on proteinuria were only available in 29 patients with active rejection and 56 patients without active rejection.

The bold values reflect significance.

### Donor-Derived Cell-Free DNA at Time of Biopsy

**FIGURE 3:**
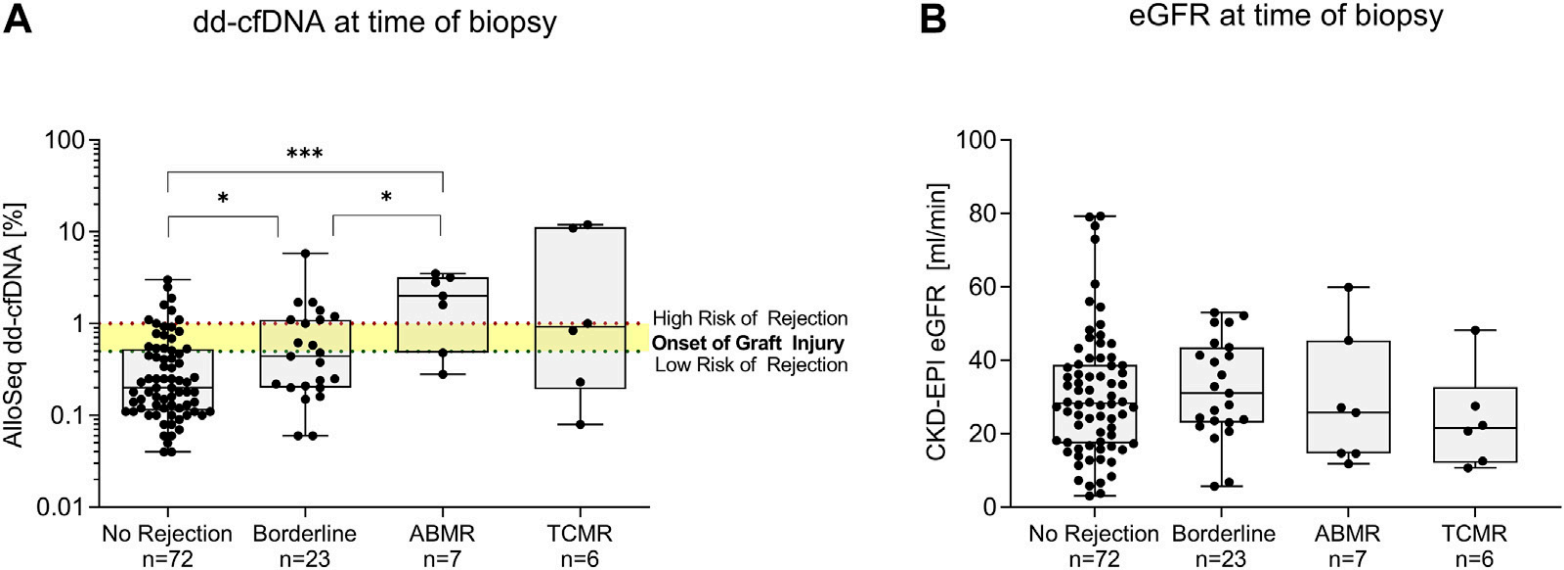
Donor-derived cell-free DNA (dd-cfDNA) and estimated glomerular filtration rate at time of biopsy. **(A)** Donor-derived cell-free DNA was highest in patients with antibody-mediated rejection, compared to patients with T cell-mediated rejection, borderline changes, and patients with no histopathological signs of rejection. The x-axis displays the respective group, dd-cfDNA levels are shown log-transformed on the y-axis. Box plots display the distribution of data, with a horizontal line denoting the median. The bottom and top edges of the box indicate the 25th and 75th percentiles respectively. Individual results are shown as dots. The red dotted line indicates a dd-cfDNA level of 1%, whereas the green dotted line indicates a dd-cfDNA level of 0.5%, corresponding to different cut-points defined in other studies investigating dd-cfDNA as a biomarker for allograft injury. Below the level of 0.5%, the risk of rejection is low. **(B)** Estimated glomerular filtration rate at time of biopsy did not differ significantly between patients with any type of rejection and no rejection. The x-axis displays the respective group, eGFR is shown on the y-axis. Box plots display the distribution of data, with a horizontal line denoting the median. The bottom and top edges of the box indicate the 25th and 75th percentiles respectively. Individual results are shown as dots. ABMR, antibody-mediated rejection; dd-cfDNA, donor-derived cell-free DNA; eGFR, estimated glomerular filtration rate; N, number; TCMR, T cell-mediated rejection; ***p < 0.001; *p < 0.05.

Patients with histopathological signs of active rejection had significantly higher levels of dd-cfDNA at time of biopsy than patients without signs for rejection, whereas estimated glomerular filtration rate (eGFR) did not differ significantly between the two groups (*p* < 0.001 and *p* > 0.99, respectively; Table 1). The diagnosis of active rejection remained independently associated with higher dd-cfDNA levels when stratified for age, gender, BMI, time since transplantation, eGFR, and the presence of donor-specific HLA or non-HLA antibodies (β: −1.071; 95% CI: −1.811, −0.331; *p* = 0.005; Supplementary Table S2).

dd-cfDNA levels were with a median (IQR) % of 2.00 (0.48–3.20) highest in patients with ABMR, followed by 0.92 (0.19–11.25) in patients with TCMR, 0.44 (0.20–1.10) in patients with borderline changes and 0.20 (0.11–0.53) in patients with no signs of rejection (Figure 3A). Patients with ABMR had significantly higher dd-cfDNA levels compared to both patients without signs of rejection or those with borderline changes (*p* < 0.001 and *p* < 0.05, respectively, Figure 3A). dd-cfDNA levels in patients with borderline changes were also significantly higher compared to patients without rejection (*p* < 0.05, Figure 3A). In contrast, eGFR did not differ significantly between the four groups (Figure 3B).

**FIGURE 4:**
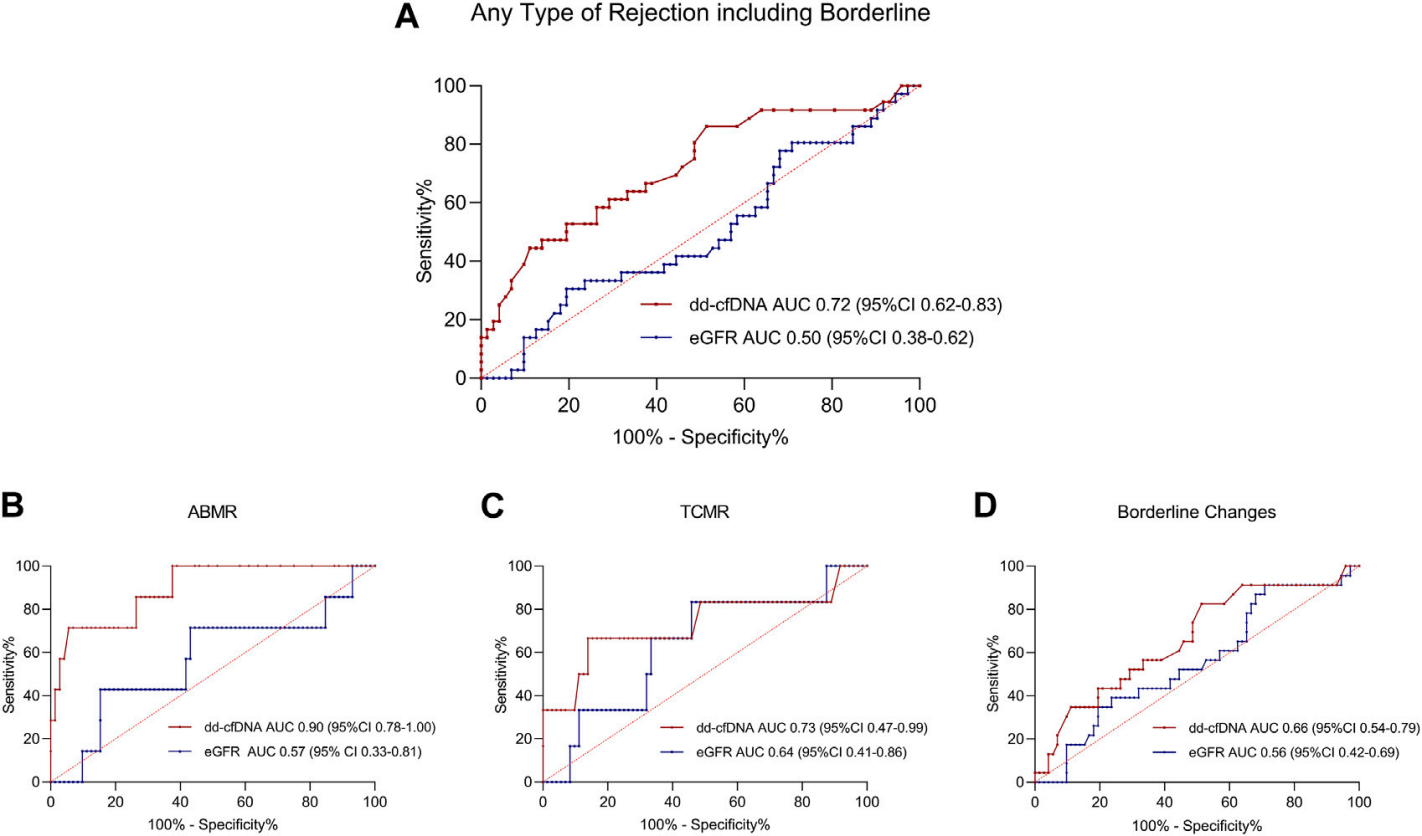
ROC Curves for donor-derived cell-free DNA and estimated glomerular filtration rate to discriminate different types of rejection from no rejection at time of biopsy. ROC curves to discriminate **(A)** any type of rejection, including borderline changes, **(B)** antibody-mediated rejection, **(C)** T cell-mediated rejection, and **(D)** borderline changes from no rejection.100%-specificity in % is displayed on the x-axis, sensitivity in % on the y-axis. dd-cfDNA is plotted in red, whereas the ROC curve for eGFR is plotted in blue for all ROC curves. Respective AUC and 95% CI is given in red for dd-cfDNA and in blue for eGFR at the bottom of each graph. ABMR, antibody-mediated rejection; AUC, area under the curve; CI, confidence interval; dd-cfDNA, donor-derived cell-free DNA; eGFR, estimated glomerular filtration rate; ROC, receiver operating characteristics; TCMR, T cell-mediated rejection.

To evaluate the diagnostic performance of dd-cfDNA to discriminate acute rejection from no rejection, the area under the ROC curve (AUC) was calculated. The AUC to discriminate any type of rejection including borderline changes from no rejection was at 0.72 (95% CI 0.62–0.83; Figure 4A). For the discrimination of only ABMR or only TCMR from no rejection, dd-cfDNA exhibited an AUC of 0.90 (95% CI 0.78–1.00, Figure 4B) and 0.73 (95% CI 0.47–0.99, Figure 4C), respectively. When only borderline changes vs. no rejection were compared, a lower AUC of 0.66 (95% CI 0.54–0.79, Figure 4D) was observed.

The optimal cut point for dd-cfDNA to discriminate active rejection from no rejection as calculated by the Youden index was at a threshold of 0.57, yielding a specificity of 81% (95% CI 70%–88%), a sensitivity of 53% (95% CI 37%–68%), a PPV of 58% (95% CI 41%–73%), and an NPV of 77% (95% CI 67%–85%). Supplementary Figure S1 displays the values of specificity and sensitivity for different measurements of dd-cfDNA to discriminate acute rejection from no rejection. Table 2 illustrates the specificity, sensitivity, PPV and NPV when applying different dd-cfDNA levels as established in other studies to our study cohort [7–9].

**TABLE 2:** Application of suggested cut points of dd-cfDNA levels in our study cohort of kidney transplant recipients with indication biopsy.

Cut point	Specificity	Sensitivity	Positive predictive value	Negative predictive value
dd-cfDNA ≥ 0.5% [9]	74% (95% CI 62%–82%)	53% (95% CI 37%–68%)	50% (95% CI 35%–65%)	76% (95% CI 65%–84%)
**dd-cfDNA ≥ 0.57%**	**81% (95% CI 70%–88%)**	**53% (95% CI 37%–68%)**	**58% (95% CI 41%–73%)**	**77% (95% CI 67%–85%)**
dd-cfDNA ≥ 0.74% [8]	82% (95% CI 72%–89%)	48% (95% CI 32%–63%)	57% (95% CI 39%–73%)	76% (95% CI 65%–84%)
dd-cfDNA ≥ 1% [7]	89% (95% CI 80%–94%)	44% (95% CI 30%–60%)	67% (95% CI 47%–82%)	76% (95% CI 66%–84%)

In literature, different cutoffs have been proposed for determining when to assume graft injury and/or rejection. Stites et al. found a 0.5% threshold of dd-cfDNA to be associated with increased risk of eGFR decline, DSA development and future episodes of rejection in patients with borderline and 1A T cell-mediated rejection [9]. Huang et al. introduced a threshold of ≥0.74% for distinguishing between cell-mediated, antibody-mediated, and mixed rejection from cases with no rejection [8]. Of note, similar to Bloom et al., who advocated for a 1% cut-off, they also excluded patients with borderline lesions from their rejection cohort [7, 8].

The bold values indicate the cut-off calculated in our study and the respective sens/Spec/PPV/NPV in contrast to other studies.

Twenty-four (22%) patients had dd-cfDNA levels ≥1%, of whom 8 had no histopathological signs of rejection. These patients were diagnosed with BKVAN (*N* = 1), acute tubular injury (ATI; *N* = 2; 8 and 11 days after living donor kidney transplantation), IFTA (*N* = 3, whereof one patient with presence of DSA), or CNI toxicity (*N* = 2, whereof 1 patient with presence of DSA). Supplementary Figure S2 displays levels of dd-cfDNA in patients with histopathological diagnoses other than rejection.

### Donor-Derived Cell-Free DNA in Patients With Borderline Changes

dd-cfDNA levels varied considerably among patients with borderline changes, ranging from 0.06% to 5.80% (Supplementary Figure S3A). When categorizing patients with borderline changes based on their dd-cfDNA levels at time of biopsy (either < or ≥ 1% and < or ≥ 0.5%), those with lower dd-cfDNA levels displayed a tendency toward an improvement in eGFR after corticosteroid pulse therapy, in contrast to patients with higher dd-cfDNA levels who exhibited relatively stable or decreasing eGFR over time, albeit not reaching statistical significance (Supplementary Figure S3B).

### Correlation of Donor-Derived Cell-Free DNA to BANFF Lesion Scores

When calculating the relationship between levels of dd-cfDNA to BANFF lesion scores, a significant moderate correlation for dd-cfDNA was established to ptc (44 patients with ptc ≥ 1; Spearman’s rho = 0.34, *p* < 0.001), and to C4d positivity (4 patients with C4d ≥ 1; Spearman’s rho = 0.30, *p* = 0.002), and a weak correlation to cg (12 patients with cg ≥ 1; Spearman’s rho = 0.21, *p* = 0.03) and to PVI (13 patients with PVI ≥ 1; Spearman’s rho = −0.26, *p* = 0.009) (Supplementary Table S3). The presence of DSA at either cutoff (MFI > 500 or MFI > 1,000) was not significantly associated with higher dd-cfDNA levels, neither was the presence of non-HLA antibodies (Spearman’s rho of −0.19, −0.14, 0.11, respectively). Higher sCD30 levels as a marker of an activated immune system were weakly but significantly associated with higher dd-cfDNA levels (Spearman’s rho = 0.2; *p* = 0.04). In the presence of DSA, the AUC for discriminating active rejection including borderline changes from no rejection with the help of dd-cfDNA was 0.77 (95% CI 0.60–0.94) when applying a cutoff of MFI > 500 and 0.75 (95% CI 0.53–0.97) when applying a cutoff of MFI>1,000 for determining DSA (Supplementary Figure S4).

### Changes in Donor-Derived Cell-Free DNA Upon Treatment

**FIGURE 5:**
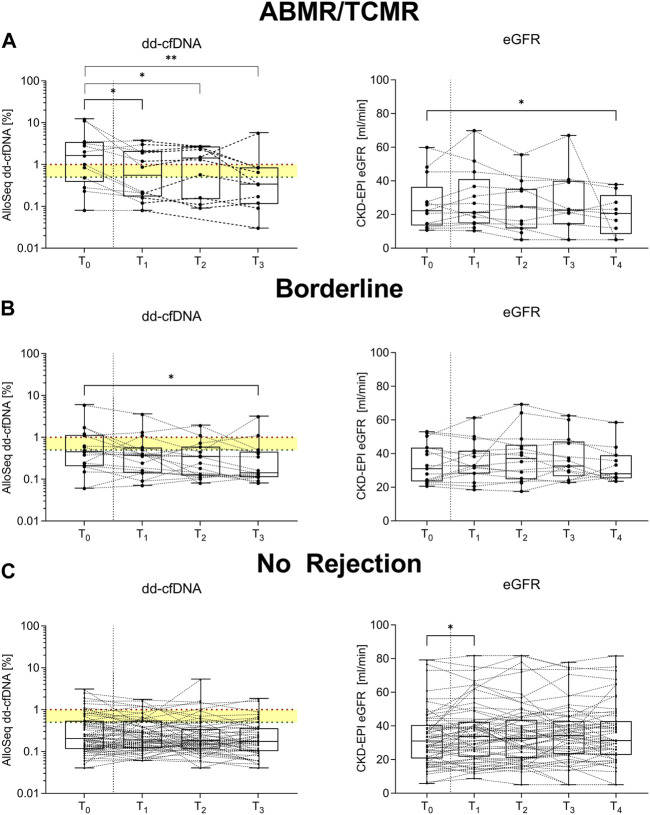
Longitudinal changes in donor-derived cell-free DNA and estimated glomerular filtration rate. **(A)** In patients with antibody-mediated or T cell-mediated rejection (*N* = 13), donor-derived cell-free DNA (dd-cfDNA) decreased comparing levels from time at biopsy (T_0_) to 7 days (T_1_), 30 days (T_2_) and 90 days (T_3_) post-biopsy. Estimated glomerular filtration rate (eGFR) remained largely unchanged. **(B)** For patients with borderline changes who received corticosteroid pulse therapy (*N* = 19), dd-cfDNA decreased significantly comparing levels at time of biopsy (T_0_) to levels 90 days post-biopsy (T_3_), whereas no significant differences were seen in eGFR when comparing values obtained at 7 (T_1_), 30 (T_2_), 90 (T_3_), and 180 days (T_4_) follow-up to eGFR at biopsy (T_0_). **(C)** No significant differences in dd-cfDNA levels were observed in patients with no histopathological signs of rejection (*N* = 72), whereas eGFR improved slightly in these patients comparing eGFR at biopsy (T_0_) to levels at 7 days post-biopsy (T_1_). The x-axis displays the respective time point, dd-cfDNA levels are shown log-transformed and eGFR is displayed linearly on the y-axis. Box plots display the distribution of data, with a horizontal line denoting the median. The bottom and top edges of the box indicate the 25th and 75th percentiles, respectively. Individual results are shown as dots. The red dotted line indicates a dd-cfDNA level of 1%, whereas the green dotted line indicates a dd-cfDNA level of 0.5%, corresponding to different cut-points defined in other studies investigating dd-cfDNA as a biomarker for allograft injury. Below the dd-cfDNA level of 0.5%, the risk of rejection is low. ABMR, antibody-mediated rejection; dd-cfDNA, donor-derived cell-free DNA; CKD-EPI eGFR, Chronic Kidney Disease Epidemiology Collaboration estimated glomerular filtration rate; TCMR, T cell-mediated rejection. **p < 0.01; *p < 0.05.

In patients with histopathological signs of ABMR or TCMR, dd-cfDNA decreased significantly when comparing levels at time of biopsy to levels at 7, 30, and 90 days of follow-up (*p* = 0.04, *p* = 0.02, and *p* = 0.002, respectively; Figure 5A). For patients with borderline changes who received corticosteroid pulse therapy (*N* = 19), dd-cfDNA decreased significantly from a median of 0.4% (0.2–1.1) at time of biopsy to 0.1% (0.1–0.4) 90 days post-biopsy (*p* = 0.03), whereas no significant differences were seen in eGFR when comparing values obtained at 7, 30, 90, and 180 days follow-up to eGFR at biopsy (Figure 5B). No significant differences in dd-cfDNA levels were observed in patients without histopathological signs of rejection, whereas eGFR improved slightly in these patients from a median (IQR) of 31.9 mL/min/1.73 m^2^ (21.3–40.5) at biopsy to 33.8 (21.8–42.4) 7 days post-biopsy (*p* = 0.04; Figure 5C). Supplementary Tables S4, S5 summarize the changes in dd-cfDNA and eGFR post biopsy, respectively, when analyzing pairs.

## Discussion

In this prospective study, we assessed the diagnostic usefulness of dd-cfDNA in a cohort of German kidney transplant recipients with indication biopsy. We found that dd-cfDNA levels were significantly higher in patients with active rejection compared to patients with no rejection. dd-cfDNA discriminated active rejection (including borderline changes diagnosed during allograft dysfunction) from no rejection with an AUC of 0.72. This is in line with results of Bloom et al. who validated dd-cfDNA in the DART study and found an AUC of 0.74 to discriminate between biopsy showing any rejection (ABMR or TCMR) vs. no histopathological signs of rejection [7]. When excluding borderline changes from the analysis, the AUC for dd-cfDNA to discriminate ABMR or TCMR from no rejection even reached a higher 0.82, albeit including only a small sample size of 13 patients. Huang et al., who reported on their early clinical experience using dd-cfDNA since it became Medicare reimbursable in the United States in October 2017, reported exactly the same AUC of 0.82 for dd-cfDNA to effectively distinguish ABMR from no rejection [8]. Thus, it appears that dd-cfDNA performs particularly well in correctly identifying active ABMR which corresponds to our findings of a significant correlation between increased dd-cfDNA levels to ptc lesion score, matching with findings of Gielis et al. [20]. Since glomerulitis (g) and intimal arteritis (v) were infrequently observed within our cohort, statistical analyses could not be performed for these specific lesions.

Next, we identified that a dd-cfDNA level of 0.57% was best to distinguish rejection (including borderline changes) from no rejection, yielding a specificity of 81%, a sensitivity of 53%, a PPV of 58%, and an NPV of 77%. The cutoffs ≥1.0% and ≥0.74%, as suggested by Bloom et al. [7] and Huang et al. [8], discriminated active rejection from no rejection in our study with specificities of 89% and 82% and sensitivities of 44% and 48%, respectively. It is evident that specificity increases at higher dd-cfDNA thresholds, however, if we used a cutoff of ≥1%, we would have misinterpreted as many as 56% of the 36 patients (2 patients with ABMR, 3 patients with TCMR, and 15 patients with borderline changes) as having no rejection when relying only on the dd-cfDNA levels. It is crucial to highlight that unlike Bloom et al. and Huang et al. we also incorporated patients with borderline lesions into the rejection group which may account for the lower sensitivity and NPV observed at our calculated 0.57% threshold [7, 8]. Specifically, 13 out of 23 (57%) patients with borderline lesions had dd-cfDNA levels below this cut-off and were thus “false negative.” In addition, a significant proportion of patients in our study were biopsied at later stages post-transplantation, revealing chronic lesions that were previously shown to be associated with lower dd-cfDNA levels, further impeding sensitivity to correctly identify rejection [21].

On the contrary, 8/24 (33%) patients with dd-cfDNA levels of ≥1% had no histopathological signs of rejection but other causes of graft injury, such as ATI, BKVAN, IFTA, or CNI-Toxicity. Regarding higher levels of dd-cfDNA in patients with no molecular or histologic rejection, Halloran et al. argued that dd-cfDNA may also be released if parenchymal injury is present, such as in acute injury or atrophy fibrosis [21]. The substantial number of patients exhibiting dd-cfDNA levels ≥1% without corresponding histopathological findings for rejection thus emphasizes that dd-cfDNA best serves as an indicator of active graft injury. Evidently, dd-cfDNA cannot differentiate the various causes of acute kidney injury following transplantation, some of which may require opposing treatment approaches. However, as stated by Roy Bloom before, it seems rather unlikely that one biomarker will emerge as a universal solution for diagnosing all kidney transplant-related issues with both high sensitivity and specificity [22]. A more practical approach would involve utilizing a combination of blood and urine biomarkers alongside various clinical parameters to provide comprehensive insights into cellular damage and immune responses [22]. Nonetheless, the expanding body of literature on dd-cfDNA underscores its potential in assisting with the identification of at-risk patients in routine clinical practice.

Another potential benefit of dd-cfDNA lies in its ability to identify patients with rejection in whom injury does not resolve upon corticosteroid pulse therapy, warranting closer monitoring, re-biopsies, and possibly more aggressive therapeutic interventions. This hypothesis is supported by Stites et al. who found that higher levels of dd-cfDNA identified patients with TCMR 1A rejection and borderline changes with more unfavorable clinical outcomes, such as eGFR decline, *de novo* DSA development, and future or persistent rejection [9]. In our study, we observed considerable variability in dd-cfDNA levels among patients with borderline changes (Supplementary Figure S3). When we categorized these patients into two groups based on their dd-cfDNA levels at the time of biopsy (either <0.5/1% or ≥0.5/1%) and compared their eGFR trajectories, we observed a tendency towards eGFR improvement in patients with lower dd-cfDNA levels whereas patients with higher dd-cfDNA levels showed stable or declining eGFR, although we could not establish statistical significance. When interpreting these findings, one should consider the controversially discussed pathological relevance of borderline changes. Borderline changes with low dd-cfDNA levels may represent non-pathogenic histological findings that may require no treatment at all. However, this hypothesis is to be tested in future studies.

In addition to helping the clinician to identify patients at risk for rejection or with severe injury, dd-cfDNA may also be of use to assess response to therapy. In agreement with the findings of Wolf-Doty et al. and Hinojosa et al., we observed decreasing levels of dd-cfDNA in patients receiving anti-rejection therapy [23, 24]. However, similar to Wolf-Doty et al., we did not observe any significant changes in eGFR or serum creatinine following treatment [23]. It is important to exercise caution when interpreting these findings as dd-cfDNA primarily serves as an indicator of injury, whereas eGFR reflects graft function. Since we did not routinely conduct re-biopsies, it remains uncertain whether the injury completely resolved with therapy, which is a limitation to our study.

Another limitation of our study is the relatively small number of cases with ABMR (*N* = 7) or TCMR (*N* = 6). However, despite this limitation, our findings align consistently with current literature, supporting the robustness and reliability of the results.

In conclusion, our prospectively designed study verified the good performance of dd-cfDNA to discriminate kidney transplant recipients with active rejection, particularly patients with ABMR, from those with histopathological findings other than rejection. Based on our results, we hypothesize that dd-cfDNA may aid the clinician in monitoring patients at risk, for example, those with *de novo* DSA or previous biopsy-proven rejection, where elevated or increasing dd-cfDNA levels may aid in decision-making regarding the necessity and timing of a graft biopsy. The potential benefit of dd-cfDNA in the assessment of response to therapy and for risk stratification of patients with borderline changes needs further validation. Additionally, it is yet to be determined whether screening with dd-cfDNA will significantly reduce the number of unnecessary biopsies and can be carried out cost-effectively.

## Data Availability

The raw data supporting the conclusion of this article will be made available by the authors, without undue reservation.
