# Supplementary material for: Donor-Derived Cell-Free DNA (dd-cfDNA) in Kidney Transplant Recipients With Indication Biopsy—Results of a Prospective Single-Center Trial
Source: Transpl Int. 2023 Nov 3;36:11899. doi: 10.3389/ti.2023.11899 (PMC10654198; doi:10.3389/ti.2023.11899)
Supplement: Supplementary file 1 [file DataSheet1.docx]

Supplementary Material

# Table of contents

Supplementary Figure S1

Sensitivity (%) and specificity (%) for dd-cfDNA to discriminate active rejection versus no active rejection in a cohort of kidney transplant recipients with indication biopsy.

Supplementary Figure S2

Levels of dd-cfDNA in patients with a histopathological diagnosis other than rejection.

Supplementary Figure S3

Levels of dd-cfDNA in patients with borderline changes.

Supplementary Figure S4

AUC for discriminating active rejection including borderline changes from no rejection with the help of dd-cfDNA in the presence of donor-specific anti-HLA antibodies (DSA).

Supplementary Table S1

Subcategories of ABMR and TCMR with respective dd-cfDNA levels.

Supplementary Table S2

Multiple linear regression analysis for possible confounders for elevated dd-cfDNA levels.

Supplementary Table S3

Correlation of dd-cfDNA levels with histopathological lesion scores according to the BANFF classification and the polyomavirus-associated interstitial nephritis score.

Supplementary Table S4

Changes in dd-cfDNA post-biopsy.

Supplementary Table S5

Changes in eGFR post-biopsy.

Supplementary Figure S1 Sensitivity (%) and specificity (%) for dd-cfDNA to discriminate active rejection versus no active rejection in a cohort of kidney transplant recipients with indication biopsy.


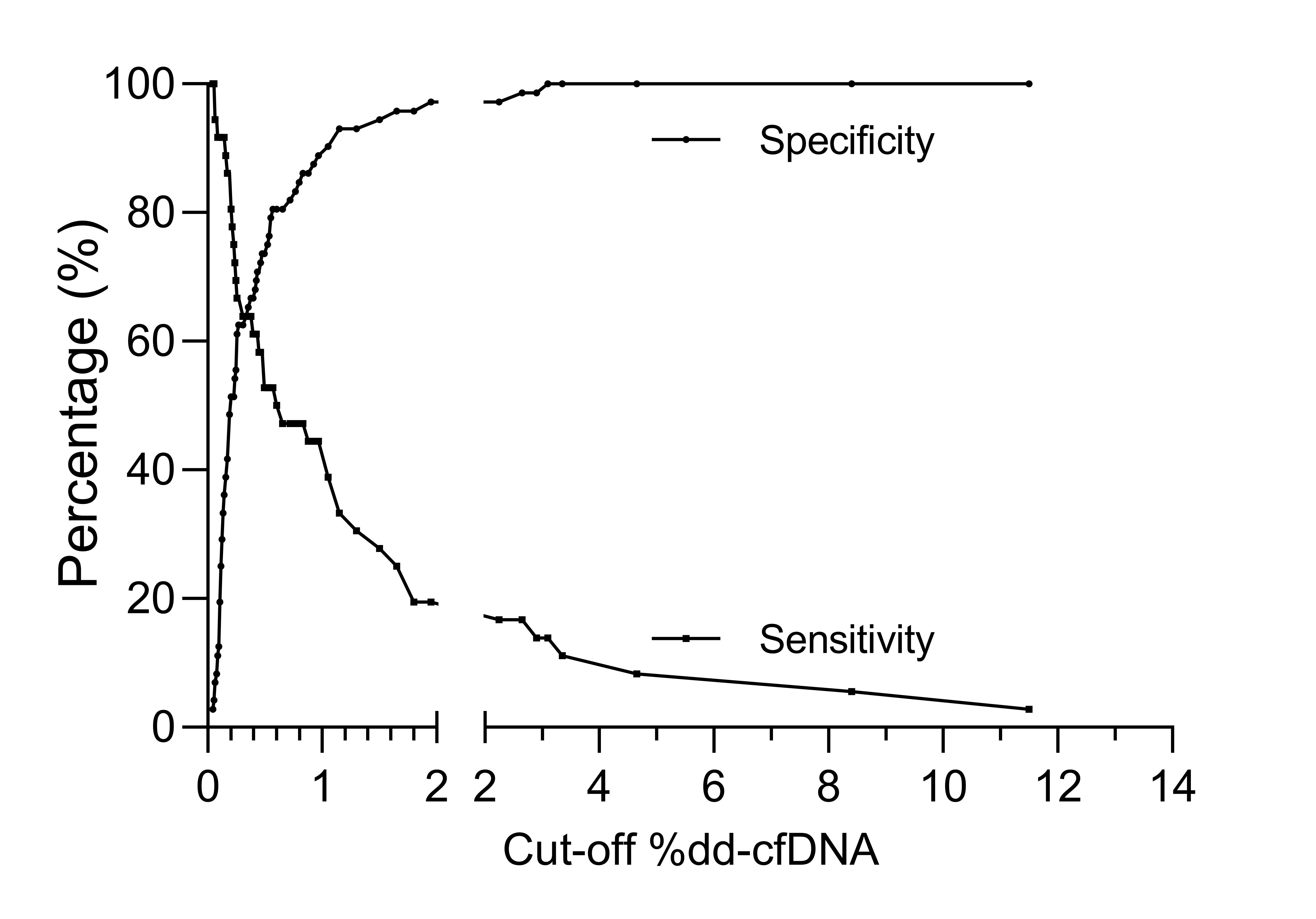


The cut-off % of donor-derived cell-free DNA is displayed on the x-axis, the percentage on the y-axis. dd-cfDNA, donor-derived cell-free DNA.

Supplementary Figure S2 Levels of dd-cfDNA in patients with a histopathological diagnosis other than rejection.


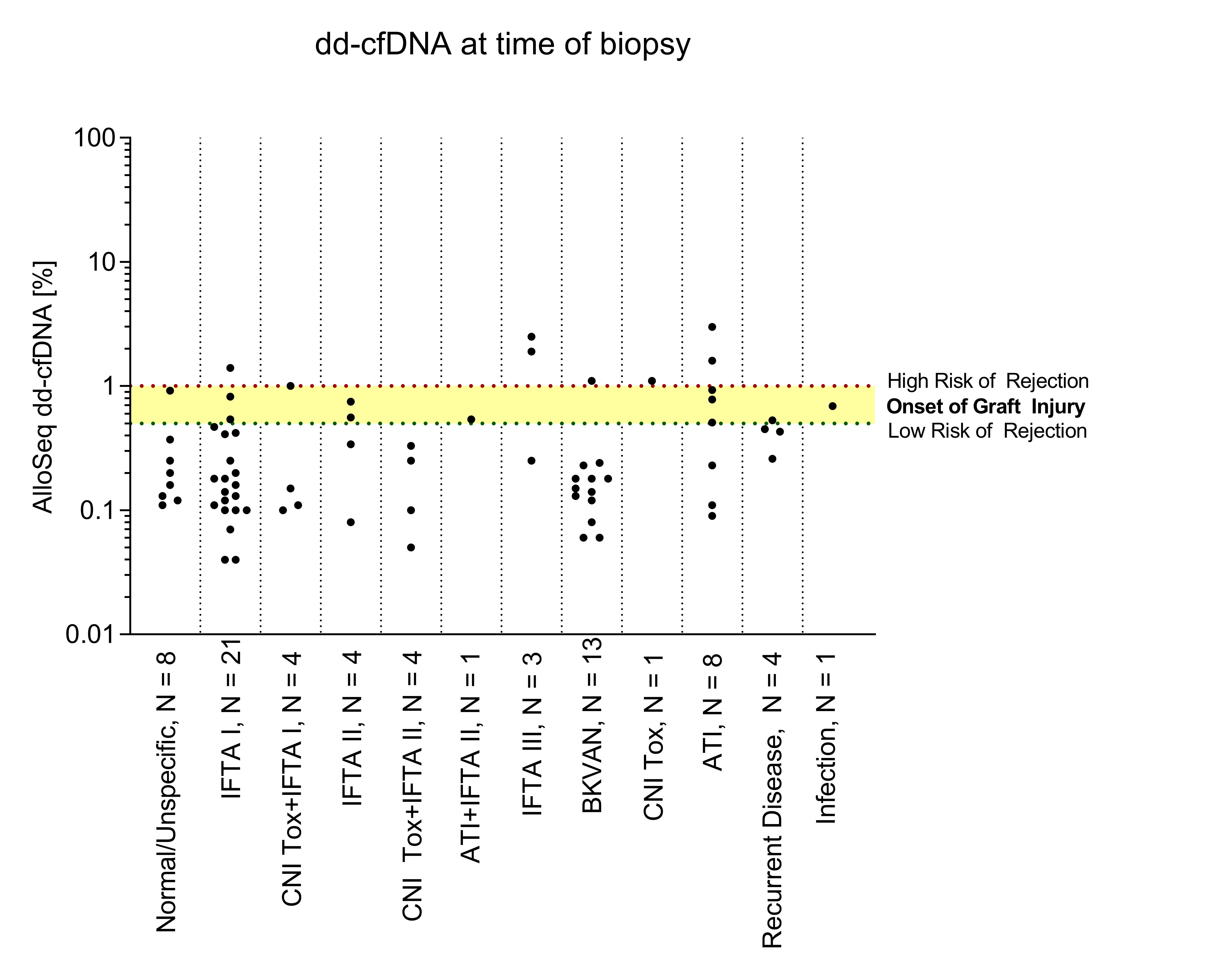


Donor-derived cell-free DNA in patients with histopathological diagnosis other than rejection at time of biopsy. The x-axis displays the respective group, dd-cfDNA levels are shown log-transformed on the y-axis. Box plots display the distribution of data, with a horizontal line denoting the median. The bottom and top edges of the box indicate the 25th and 75th percentiles respectively. Individual results are shown as dots. The red dotted line indicates a dd-cfDNA level of 1%, whereas the green dotted line indicates a dd-cfDNA level of 0.5%, corresponding to different cut-points defined in other studies investigating dd-cfDNA as a biomarker for allograft injury. Below the level of 0.5%, the risk of rejection is low. ATI, acute tubular injury; BKVAN, BK virus-associated nephropathy; CNI Tox, calcineurin inhibitor toxicity; dd-cfDNA, donor-derived cell-free DNA; IFTA, interstitial fibrosis and tubular atrophy; N, number.

Supplementary Figure S3 Levels of dd-cfDNA in patients with borderline changes.





(A) Patients with borderline changes exhibited considerable variation in % of dd-cfDNA upon biopsy, ranging from 0.06% to 5.80%. (B) When categorizing patients with borderline changes based on their dd-cfDNA levels at time of biopsy (either < or ≥ 1%, left panel, and < or ≥ 0.5%, right panel), those with lower dd-cfDNA levels displayed a tendency toward an improvement in eGFR with time, in contrast to patients with higher dd-cfDNA levels who exhibited relatively stable or decreasing eGFR with time, albeit not reaching statistical significance.

Supplementary Figure S4 AUC for discriminating active rejection including borderline changes from no rejection with the help of dd-cfDNA in the presence of donor-specific anti-HLA antibodies (DSA).


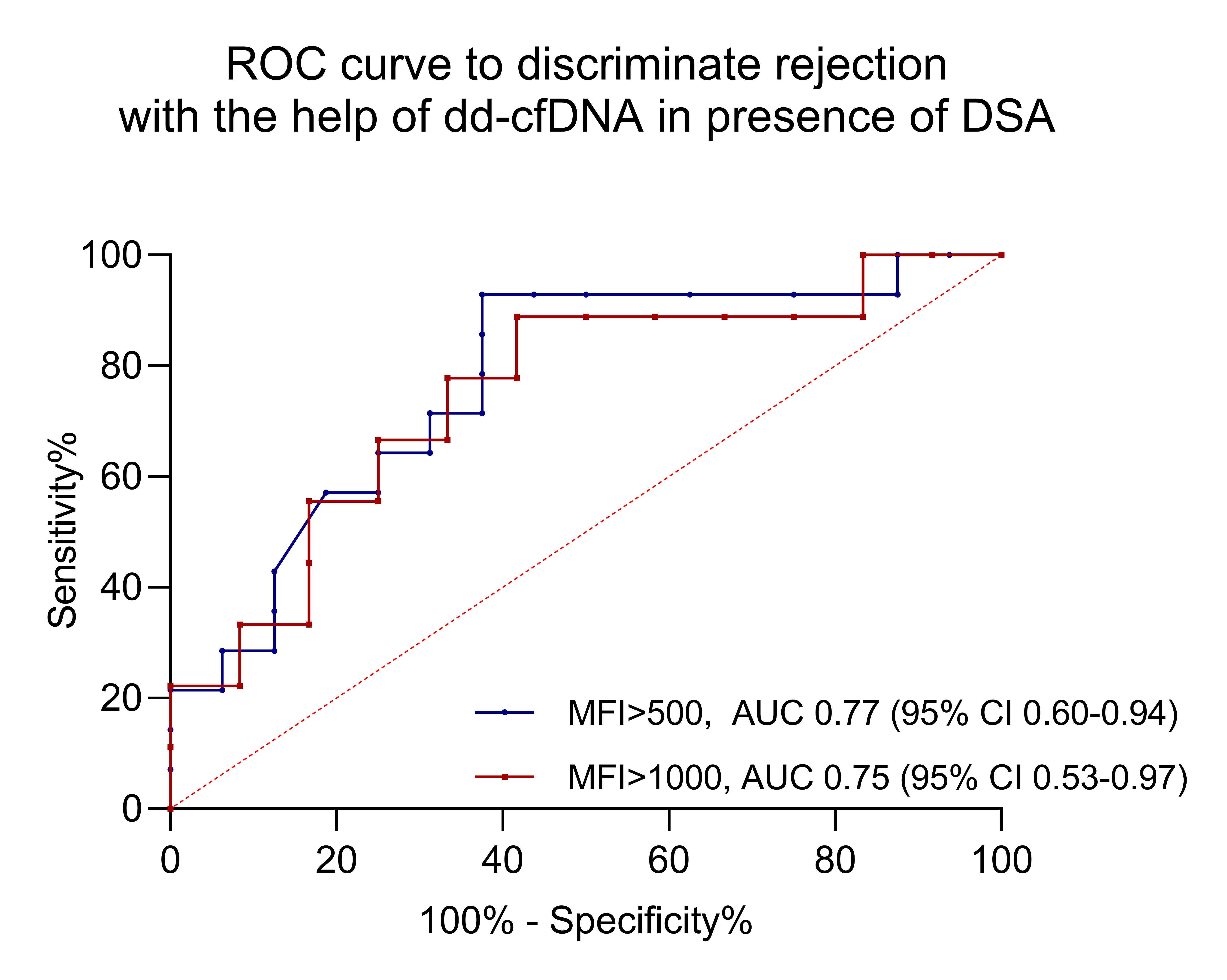


(A) ROC curve to discriminate rejection from no rejection in the presence of DSA with different cuf-offs (MFI>500 and MFI>1000). 100%-specificity in % is displayed on the x-axis, sensitivity in % on the y-axis. AUC, area under the curve; CI, confidence interval; dd-cfDNA, donor-derived cell-free DNA; DSA, donor-specific HLA antibodies ; MFI, mean fluorescence intensity; ROC, receiver operating characteristics.

Supplementary Table S1 Subcategories of ABMR and TCMR with respective dd-cfDNA levels.

| **Patientt** | **Biopsy** | **S-Crea** | **eGFR** | **dd-cfDNA** | **donor-specific anti-HLA antibody (DSA)** | **MICAA** | **AT1-R** | **ETA** | **S-**  **CD30** |
| --- | --- | --- | --- | --- | --- | --- | --- | --- | --- |
| ABMR 1 | Chronic Active ABMR  (i1, t0, g0, ptc2, C4d3, ci1, ct1, mm3, ah2, i-IFTA1, pVI0) | 4,7 | 14,7 | 3,5 | max.MFI:25842 | 0 | 8 | 7 | 71 |
| ABMR 2 | Chronic Active ABMR  (i1, t1, v0, g0, ptc2, C4d0, ci1, ct1, cv1, cg2, mm1, ah2, i-IFTA1, pVI0) | 3,12 | 25,8 | 3,2 | No assessment of DSA possible, TX in India, no Donor/Recipient Data | 0 | 8 | 9 | 82 |
| ABMR 3 | Active ABMR  (i1, t1, v0, g0, ptc2, C4d0, ci1, ct1, cv1, cg3, mm1, ah2, i-IFTA1, pVI0) | 2,84 | 27,1 | 2,8 | max.MFI:11982 | 0 | 6 | 7 | 32 |
| ABMR 4 | Chronic Active ABMR  (i1, t0, v0, g0, ptc2, C4d1, ci3, ct1, cv1, cg3, ah2, i-IFTA1, pVI0) | 3,4 | 14,6 | 2 | No DSA | 0 | 12 | 13 | 24 |
| ABMR 5 | Chronic ABMR  (i1, t0, g0, ptc1, C4d1, ci1, ct1, cg0, mm0, ah3, i-IFTA1, pVI0) | 1,69 | 45,4 | 1,6 | max.MFI:22309 | 0 | 9 | 7 | 12 |
| ABMR 6 | Chronic Active ABMR  (i1, t0, v0, g0, ptc1, C4d0, ci1, ct1, cv1, cg3, ah2, i-IFTA1, pVI0) | 1,26 | 59,9 | 0,48 | max.MFI:26030 | 0 | 9 |  | 21 |
| ABMR 7 | Chronic Active ABMR  (i1, t0, v0, g0, ptc2, C4d0, ci3, ct2, cv2, cg3, mm1, ah2, i-IFTA1) | 5,94 | 11,8 | 0,28 | max.MFI: 26973 | 0 | 7 | 10 | 48 |
| TCMR 1 | TCMR 2A  (i3, t3, v1, g0, ptc1, C4d0, ci0, ct0, cg0, mm0, ah0, pVI0) | 1,55 | 48,2 | 12 | No DSA | 60 | 7 | 7 | 170 |
| TCRM 2 | TCMR 1B  (i3, t3, v0, g0, ptc1, C4d0, ci0, ct0, cv1, cg0, mm0, ah1, pVI0) | 2,43 | 27,5 | 11 | max.MFI:4162 | 0 | 6 | 6 | 31 |
| TCRM 3 | TCMR 1B  (i3, t3, v0, g0, ptc0, C4d0, ci0, ct1, cv1, cg0, mm0, ah1, i-IFTA0, pVI0) | 2,94 | 22,3 | 1 | No DSA | 0 | 6 | 5 | 17 |
| TCMR 4 | Chronic Active TCMR 1A  (i1, t2, v0, g0, ptc1, C4d0, ci3, ct3, cv1, cg0, mm3, ah2, i-IFTA2, pVI0) | 5,19 | 12,5 | 0,84 | No DSA | 7 | >40 | >40 | 45 |
| TCMR 5 | Acute TCMR 1B  (i3, t3, v0, g0, C4d0, ci1, ct1, cv1, cg0, mm1, ah2, i-IFTA3, pVI0) | 2,86 | 20,7 | 0,23 | No DSA | 0 | 10 | 11 | 129 |
| TCMR 6 | Acute TCMR 1B  (i1, t3, v0, g0, ptc0, C4d0, ci0, ct0, cv0, cg0, mm0, ah1, i-IFTA0) | 4,1 | 10,7 | 0,08 | No DSA | 0 | 7 | 6 | 26 |

ABMR, antibody-mediated rejection; AT1R, angiotensin II type 1 receptor; dd-cfDNA, donor-derived cell-free DNA; DSA, donor-specific antibodies; eGFR, estimated glomerular filtration rate; ETA, endothelin receptor subtype A; MFI, mean fluorescence intensity; MICA, major histocompatibility complex class I-related chain A; sCD30, soluble CD30; TCMR, T cell-mediated rejection; Tx, transplant.

BANFF lesion scores: i, interstitial inflammation; t, tubulitis; v, intimal arteritis; g, glomerulitis; ptc, peritubular capillaritis; ci, interstitial fibrosis; ct, tubular atrophy; cv, vascular fibrous intimal thickening; cg, GBM double contours (cg); mm, mesangial matrix thickening; ah, hyaline arteriolar thickening; i-IFTA, inflammation in the area of IFTA; PVI, polyomavirus-associated interstitial nephritis score.

Supplementary Table S2 Multiple linear regression analysis for possible confounders for elevated dd-cfDNA levels.

| **Characteristic** | **B** | **95% CI** | **SE** | ***P* Value** |
| --- | --- | --- | --- | --- |
| Age at biopsy (years) | -0.011 | -0.035, 0.013 | 0.012 | 0.35 |
| Sex (female versus male) | 0.322 | -0.418, 1.061 | 0.373 | 0.39 |
| BMI (kg/m^2^) | -0.021 | -0.092, 0.050 | 0.036 | 0.56 |
| Time since TX | -0.003 | -0.008, 0.001 | 0.002 | 0.15 |
| eGFR (ml/min/1.73m^2^) | -0.002 | -0.023, 0.019 | 0.011 | 0.86 |
| Histopathological rejection (including borderline changes) | -1.071 | -1,811; -0,331 | 0.373 | 0.005^(**)^ |
| DSA MFI > 500 | -0.233 | -1.463; 0.998 | 0.620 | 0.71 |
| DSA MFI >1000 | -0.741 | -2.127, 0.646 | 0.698 | 0.29 |
| Presence of non-HLA antibodies | 0.215 | -0.501, 0.930 | 0.360 | 0.55 |

BMI, body mass index; DSA, donor-specific antibodies; CI, confidence interval of regression coefficient B; B, regression coefficient; SE, standard error; MFI, mean fluorescence intensity; TX, transplantation

Supplementary Table S3 Correlation of dd-cfDNA levels with histopathological lesion scores according to the BANFF classification and the polyomavirus-associated interstitial nephritis score.

| **Lesion** | **Spearman’s rho (95% CI)** | ***P*-Value** |
| --- | --- | --- |
| Interstitial inflammation (i) | 0.11 (-0.08–0.30) | 0.25 |
| Tubulitis (t) | 0.13 (-0.07–0.32) | 0.19 |
| Intimal arteritis (v) | NA |  |
| Glomerulitis (g) | NA |  |
| Peritubular capillaritis (ptc) | 0.34 (0.15–0.51) | <0.001 ^(***)^ |
| C4d | 0.30 (0.11–0.46) | 0.002 ^(**)^ |
| Interstitial fibrosis (ci) | 0.01 (-0.19–0.20) | 0.94 |
| Tubular atrophy (ct) | -0.01 (-0.20–0.19) | 0.95 |
| Vascular fibrous intimal thickening (cv) | 0.18 (-0.04–0.38) | 0.10 |
| GBM double contours (cg) | 0.21 (0.01–0.40) | 0.03 (*) |
| Mesangial matrix thickening (mm) | -0.06 (-0.26–0.15) | 0.57 |
| Hyaline arteriolar thickening (ah) | 0.01 (-0.19–0.21) | 0.94 |
| Inflammation in the area of IFTA (i-IFTA) | 0.03 (-0.18–0.24) | 0.79 |
| PVI | -0.26 (-0.45 – -0.06) | 0.009 ^(**)^ |

GBM, glomerular basement membrane; IFTA, interstitial fibrosis and tubular atrophy; NA, not applicable; PVI, polyomavirus-associated interstitial nephritis score; *** *P*<0.001; ***P*<0.01; **P*<0.05.

Supplementary Table S4 Changes in dd-cfDNA post-biopsy.

| Group | 7d post-biopsy (T_1_) follow-up | | | | 30d (T_2_) post-biopsy follow-up | | | | 90d (T_3_) post-biopsy follow-up | | | |
| --- | --- | --- | --- | --- | --- | --- | --- | --- | --- | --- | --- | --- |
|  | N | T_0_  Median (IQR) | T_1_  Median (IQR) | *P*-Value | N | T_0_  Median (IQR) | T_2_  Median (IQR) | *P*-Value | N | T_0_  Median (IQR) | T_3_  Median (IQR) | *P*-Value |
| BPAR | 28 | 0.6  (0.2–1.9) | 0.4  (0.1–1.2) | **0.006 ^(**)^** | 25 | 0.8  (0.3–2.4) | 0.4  (0.1–1.4) | **0.002 ^(**)^** | 26 | 0.5  (0.2–2.0) | 0.2  (0.1–0.5) | **<0.001 ^(***)^** |
| ABMR | 6 | 2.4  (0.4–3.3) | 1.7  (0.2–3.2) | 0.44 | 5 | 2.8  (1.2–3.4) | 2.6  (0.7–2.7) | 0.31 | 4 | 3  (1.0–3.4) | 0.5  (0.2–0.8) | 0.13 |
| TCMR | 6 | 0.9  (0.2–11.3) | 0.2  (0.1–1.1) | 0.06 | 5 | 1.0  (0.5–11.5) | 0.6  (0.1–1.9) | 0.06 | 6 | 0.9  (0.2–11.3) | 0.3  (0.1–2.0) | **0.03 ^(*)^** |
| Borderline | 16 | 0.4  (0.2–1.1) | 0.4  (0.1–0.6) | 0.08 | 15 | 0.5  (0.2–1.1) | 0.3  (0.1–0.6) | 0.1 | 16 | 0.4  (0.2–1.1) | 0.1  (0.1–0.4) | **0.03 ^(*)^** |
| No Rejection | 62 | 0.2  (0.1–0.5) | 0.2  (0.1–0.5) | 0.05 | 53 | 0.2  (0.1–0.5) | 0.2  (0.1–0.3) | 0.18 | 51 | 0.2  (0.1–0.6) | 0.2  (0.1–0.4) | 0.17 |

**Only pairs were analyzed.** The first column displays the respective study group. Distribution of dd-cfDNA at time of biopsy (T_0_), at follow-up 7 days post-biopsy (T_1_), at follow-up 30 days post-biopsy (T_2_), and at follow-up 90 days post-biopsy (T_3_) is given, along with the number of patients in each group with a paired dd-cfDNA measurement. *P*-values were calculated using the paired Wilcoxon-rank-sum test. Four patients were excluded from the borderline group as they did not receive corticosteroid pulse treatment due to concomitant infection.

ABMR, antibody-mediated rejection; BPAR, biopsy–proven acute rejection; dd-cfDNA, donor-derived cell-free DNA; IQR, interquartile range; TCMR, T cell–mediated rejection; *** *P*<0.001; ***P*<0.01; **P*<0.05.

Supplementary Table S5 Changes in eGFR post-biopsy.

| Group | 7d post-biopsy (T_1_) follow-up | | | | 30d (T_2_) post-biopsy follow-up | | | | 90d (T_3_) post-biopsy follow-up | | | | 180d (T_4_) post-biopsy follow-up | | | |
| --- | --- | --- | --- | --- | --- | --- | --- | --- | --- | --- | --- | --- | --- | --- | --- | --- |
|  | N | T_0_  Median (IQR) | T_1_  Median (IQR) | *P*-Value | N | T_0_  Median (IQR) | T_2_  Median (IQR) | *P*-Value | N | T_0_  Median (IQR) | T_3_  Median (IQR) | *P*-Value | N | T_0_  Median (IQR) | T_4_  Median (IQR) | *P*-Value |
| BPAR | 28 | 26.5(21.0–43.0) | 31.2(20.6–41.4) | 0.21 | 25 | 27.1(21.4–42.5) | 29.0(20.1–41.7) | 0.92 | 24 | 26.5(22.1–43.9) | 31.1(22.7–40.6) | 0.85 | 20 | 27.7(22.3–47.5) | 26.0(21.2–35.8) | **0.05^(*)^** |
| ABMR | 7 | 25.8(14.6–45.4) | 20.9(15.1–45.4) | >0.99 | 5 | 25.8(14.7–43.5) | 16.1(10.5–32.4) | 0.06 | 5 | 27.1(20.3–52.7) | 22.6(13.1–40.4) | 0.06 | 4 | 36.3(26.1–56.3) | 28.2(17.5–37.4) | 0.13 |
| TCMR | 6 | 21.5(12.1–32.7) | 26.1(13.5–45.0) | 0.06 | 6 | 21.5(12.1–32.7) | 29.3(12.1–40.3) | 0.16 | 6 | 21.5(12.1–32.7) | 22.0(12.0–46.2) | 0.56 | 5 | 20.7(11.6–37.9) | 11.9(5.0–25.2) | 0.44 |
| Borderline | 15 | 31.0(23.5–43.5) | 32.7(28.1–41.7) | 0.67 | 14 | 32.0(23.7–45.2) | 37.1(24.8–45.3) | 0.76 | 13 | 27.9(23.3–45.0) | 32.6(26.6–47.2) | 0.2 | 11 | 31.0(23.0–50.4) | 28.0(25.4–39.0) | 0.56 |
| No Rejection | 58 | 31.9(21.3–40.5) | 33.8(21.8–42.4) | **0.04 ^(*)^** | 62 | 31.1(20.2–40.5) | 32.5(21.0–43.7) | 0.35 | 61 | 31.8(21.0–40.7) | 34.3(23.4–42.9) | 0.26 | 57 | 31.8(20.0–40.7) | 31.3(22.8–42.9) | 0.19 |

**Only pairs were analyzed.** The first column displays the respective study group. eGFR at time of biopsy (T_0_), at follow-up 7 days post biopsy (T_1_), at follow-up 30 days post biopsy (T_2_), at follow-up 90 days post biopsy (T_3_), and at clinical follow-up 180d post biopsy (T_4_) is given, along with the number of patients in each group with a paired eGFR measurement. For patients returning to dialysis, an arbitrary eGFR=5 ml/min/1.73m^2^ was set. *P*-values were calculated using the paired Wilcoxon-rank-sum test. For analysis of eGFR, only patients receiving a biopsy ≥14d post transplantation were included. Four patients were excluded from the borderline group as they did not receive corticosteroid pulse treatment due to concomitant infection.

ABMR, antibody-mediated rejection; BPAR, biopsy–proven acute rejection; dd-cfDNA, donor-derived cell-free DNA; IQR, interquartile range; TCMR, T cell–mediated rejection; ***P*<0.01; **P*<0.05.
